# CYP450s-Activity Relations of Celastrol to Interact with Triptolide Reveal the Reasons of Hepatotoxicity of *Tripterygium wilfordii*

**DOI:** 10.3390/molecules24112162

**Published:** 2019-06-08

**Authors:** Chunhuan Jin, Zijun Wu, Lili Wang, Yoshikatsu Kanai, Xin He

**Affiliations:** 1 School of Traditional Chinese Medicine, Guangdong Pharmaceutical University, Panyu District, Guangzhou 510006, Guangdong, China; jin@pharma1.med.osaka-u.ac.jp; 2 Department of Bio-system Pharmacology, Graduate School of Medicine, Osaka University, Suita, Osaka 565-0871, Japan; ykanai@pharma1.med.osaka-u.ac.jp; 3 School of Chinese Materia Medica, Tianjin University of Traditional Chinese Medicine, Jinghai District, Tianjin 301617, China; lhd_zjw@163.com (Z.W.); wll1980@126.com (L.W.)

**Keywords:** celastrol, triptolide, CYP450 enzymes, hepatotoxicity, drug-drug interaction (DDI)

## Abstract

Celastrol and triptolide, as the two main bio-activity ingredients in *Tripterygium wilfordii,* have wide anticancer pharmacological potency, as well as anti-inflammatory and immunosuppression effects. However, they have potential hepatotoxicity and underlying mechanisms of them-induced toxicity mediated by hepatic CYP450s have not been well delineated. In the present study, we accessed the toxic effects and possible mechanism of celastrol and triptolide on primary rat hepatocytes. Models of subdued/enhanced activity of CYP450 enzymes in primary rat hepatocytes were also constructed to evaluate the relationship between the two ingredients and CYP450s. LC-MS/MS was used to establish a detection method of the amount of triptolide in rat hepatocytes. As the results, cell viability, biochemical index, and mitochondrial membrane potential indicated that celastrol and triptolide had toxic potencies on hepatocytes. Moreover, the toxic effects were enhanced when the compounds combined with 1-aminobenzotriazole (enzyme inhibitor) while they were mitigated when combined with phenobarbital (an enzyme inducer). Meanwhile, celastrol could affect the amount of triptolide in the cell. We therefore put forward that increase of triptolide in the cell might be one of the main causes of hepatotoxicity caused by *Tripterygium wilfordii*.

## 1. Introduction

*Tripterygii wilfordii* Radix, root of *Tripterygium wilfordii* Hook. F (Celastraceae, *Tripterygium*), is a frequently used herbal medicine in traditional Chinese medicine, and has been verified as an anti-inflammatory, immune-suppressing, contraceptive, and anti-tumor medicine by pharmacological researches [1,2]. The commercially available tripterygium preparations include *Tripterygium* polycoride tablet, *Tripterygium* bilayer tablet, *Tripterygium* tablet, and *Tripterygium* total terpenoids tablet, etc. [3]. As a non-steroid immunosuppressive agent, the *Tripterygium* polycoride tablet has been used in the clinic since 1984, and has become the most extensively used one in *Tripterygium* preparations [4]. Nevertheless, *Tripterygium* preparations have been reported to cause adverse reactions according to clinical records over the past fifty years, such as reproductive toxicity, endocrine system damage, digestive system damage, and especially, hematologic toxicity [5].

**Figure 1:**
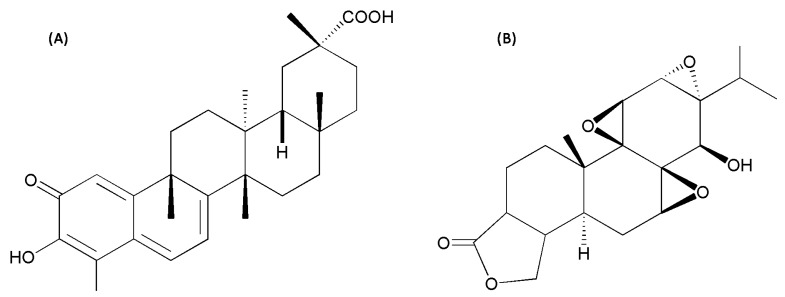
Chemical structure of two ingredients from *Tripterygii wilfordii.* (**A**) Celastrol, and (**B**) triptolide.

Celastrol (Figure 1A) is a red needle-like crystalline solid which is poorly soluble in water and soluble in most organic solvents [6]. As one of the effective compounds in anti-rheumatoid medicines, including *Tripterygii wilfordii* Radix tablets and *Tripterygium* Glycosides tablets, plenty of reports have been published on its remarkable pharmacological potency with anti-inflammation, anti-oxidation, immune-suppression, and anti-virus effects [7]. Recently, studies on celastrol have surged due to its potential anti-tumor effect [8,9,10].

“Effective with moderate dose yet toxic with excessive dose” is the chief characteristic of *Tripterygium* preparations. However, the research is significantly lagging between the pharmacodynamic/toxicodynamic material base and quality control. For example, triptolide (not more than 10 μg) and wilforlide A (not less than 10 μg) [11] are used as the quality control indicators for *Tripterygium* polycoride tablets. Although triptolide (Figure 1B) is the most active epoxy diterpene lactone in *Tripterygium wilfordii* and leads to the toxicities, its extremely low content has prevented it from reflecting the overall effects and toxicities of *Tripterygium* preparations [12]. That is, therefore, an important reason to induce clinical adverse reactions for the unclear pharmacodynamic/toxicodynamic material base of *Tripterygium* preparations.

The liver is a crucial organ in the process of metabolism and bio-transformation. In vitro, primary hepatocytes keep almost equivalent metabolism and expression of CYP450s for in vivo hepatocytes. Meanwhile, primary hepatocyte tests are reproducible and efficient enough to provide comprehensive information on drug absorption and metabolism on the cellular level [13,14]. Therefore, the in vitro primary hepatocyte system for drug metabolism is an efficient tool in drug evaluation, it defines a standard method for figuring out drug metabolism pathways, evaluating drug safety and toxicological research especially; the drug is in highly toxic or hard to detect [15,16]. FDA regards it as the gold standard of in vitro metabolic research [17]. The method is extraordinarily useful to ascertain the mechanism of triptolide and celastrol-induced hepatocellular toxicity and possible drug–drug interactions.

CYP450s are the most important Phase I metabolic enzymes. They are involved in the metabolism of both endogenous and exogenous substances and subsequently involved in drug metabolism and drug interaction [18]. Induction or inhibition of CYP450s can dramatically alter metabolic pathways, and even the efficacy and safety of drugs [19]. In the present study, five important CYP450 hypotype enzymes in the liver were selected as the objects [20]. For example, as the isomer with the largest protein expression in liver, CYP3A4 makes up about 30%–40% CYP in human liver, and metabolizes 50% of drugs in the clinical setting [21]. Ranking third place in enzyme content, CYP1A2 is mainly distributed in the liver and makes up about 13% of CYPs in the liver [22]. It takes part in the metabolism of numerous pro-toxicants and procarcinogens [23,24]. CYP2C19 has genetic polymorphisms. Although making up only 1.5% of the human metabolic enzyme [25,26], CYP2D6 metabolizes about 30% of drugs. Distributed in liver, lung, gastrointestinal tract, kidney, and brain, it mainly takes part in the metabolism of nitrosamine. CYP2D6 has been so far the metabolic enzyme with the most distinct genetic polymorphism and mainly takes part in the metabolism of nitrosamine. Mainly taking part in the metabolism of nitrosamine as well as its precursors, CYP2E1 is able to be induced by ethanol. It is mainly distributed in the adult liver and gathers in the central lobule zones. With obvious genetic polymorphisms, there are 75 varieties of substrates in CYP2E1, while the majority of them are procarcinogens and pro-toxicants and the minorities are drugs [27,28].

In this study, isolated rat primary hepatocytes were used as the model to explore the potential mechanism for celastrol and triptolide to induce hepatotoxicity. To further determine the role of CYP450 enzymes in the mechanism of the toxicity caused by these two compounds, subdued/enhanced activity CYP450 enzymes in primary rat hepatic cell models, which were treated by broad-spectrum inhibitor or inducer of CYP450s respectively, were selected to study for cell viability [25,29,30], biochemical indicators, and cell status detection. LC-MS/MS assay was used to detect the accumulative level of triptolide in hepatocytes.

## 2. Results

### 2.1. Hepatotoxicity of Celastrol and Triptolide

**Figure 2:**
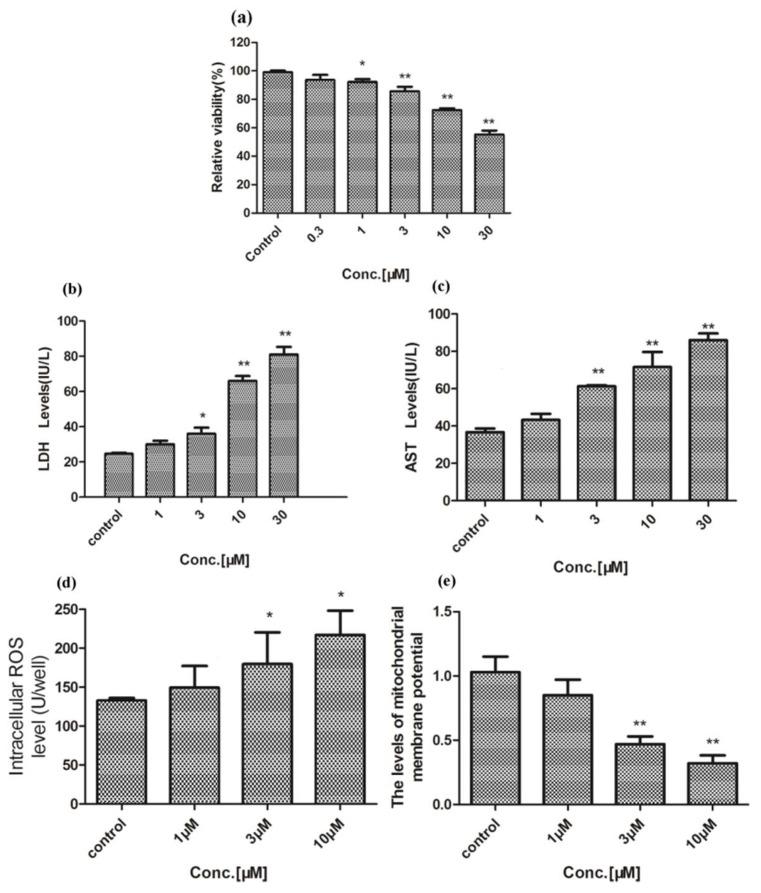
The toxicity of celastrol on primary rat hepatocytes. (**A**) The relative viability after treatment with a series of concentrations of celastrol. (**B**–**D**) The effects of celastrol on LDH, AST, and ROS productions in rat primary hepatocyte, respectively. (**E**) The MMP after administration of different concentrations of celastrol. Compared with the control cells, * *p* < 0.05, and ** *p* < 0.01.

**Figure 3:**
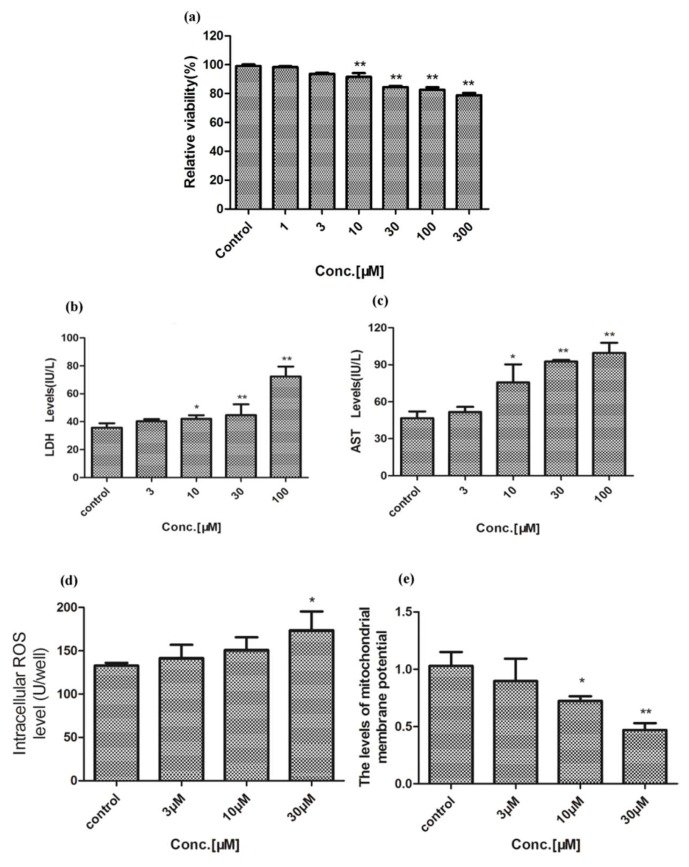
Toxicity of triptolide on primary rat hepatocytes. (**A**) The relative viability after treatment with a variety of concentrations of triptolide. (**B**–**D**) The effects of triptolide on LDH, AST, and ROS productions in rat primary hepatocyte, respectively. (**E**) MMP levels after treatment with different concentrations of triptolide. Compared with the control group, * *p* < 0.05, ** *p* < 0.01.

After administration of different concentrations of celastrol to primary rat liver cells for 24 h, XTT assay was used to determine cell viability as shown in Figure 2A. Compared with the control group, the cell viability was significantly decreased following exposure to 3, 10, and 30 μM celastrol (*p* < 0.01), and the toxic effects of different extents were shown in a dose-dependent manner. Cell damage associated with celastrol was assayed by increasing LDH and AST productions. AST and LDH productions increased in 3, 10 and 30 μM (*p* < 0.05) (Figure 2B,C). ROS production was measured by using the fluorescent dye DCFH-DA. Compared with control cells, ROS production increased significantly (*p* < 0.05) after treatment of 3 μM celastrol (Figure 2D). The low Δ*Ψm* is a sign of early apoptosis. Compared with the control group, Δ*Ψm* in primary rat hepatocyte was lower after being exposed to 3, 10 and 30 μM celastrol for 24 h (*p* < 0.01) (Figure 2E). For triptolide, results on XTT, LDH, AST, ROS, and MMP were similar to that of celastrol (Figure 3). Both of the two compounds showed the toxic effect on primary rat hepatic cells.

### 2.2. Effect of Celastrol and Triptolide on Primary Rat Hepatocytes Model with ABT

**Figure 4:**
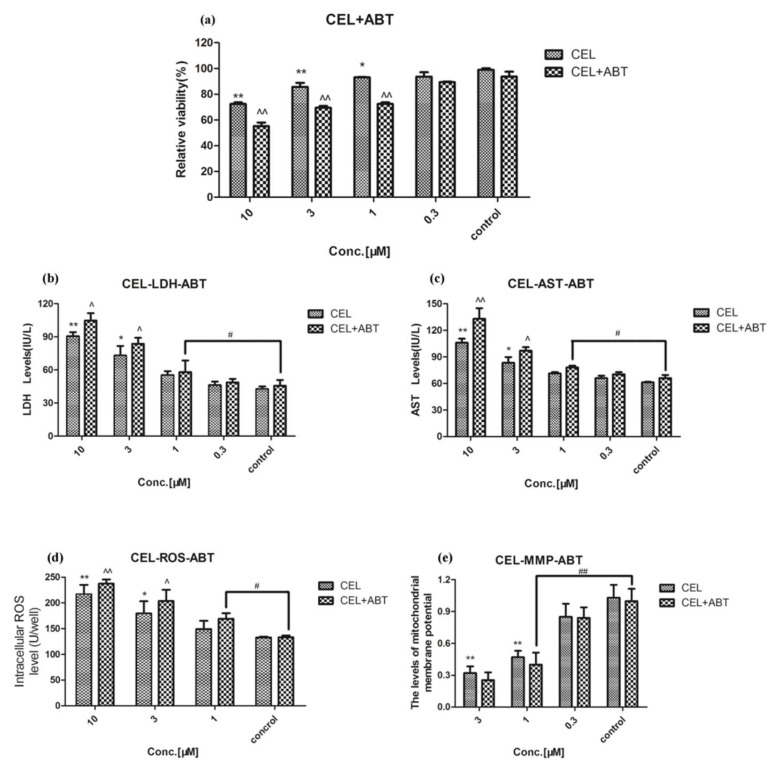
Influence of celastrol on the cell after preincubation with ABT. (**A**) The relative viability after treatment with a variety of concentrations of celastrol. (**B**–**D**) Cellular LDH, AST, and ROS productions after treatment with celastrol, respectively. (**E**) The MMP after treatment with different concentrations of celastrol. Compared with the control group, * *p* < 0.05, and ** *p* < 0.01; compared with the celastrol group, ^ *p* < 0.05, and ^^ *p* < 0.01; compared with ABT treated group, ^#^ *p* < 0.05, and ^##^ *p* < 0.01.

**Figure 5:**
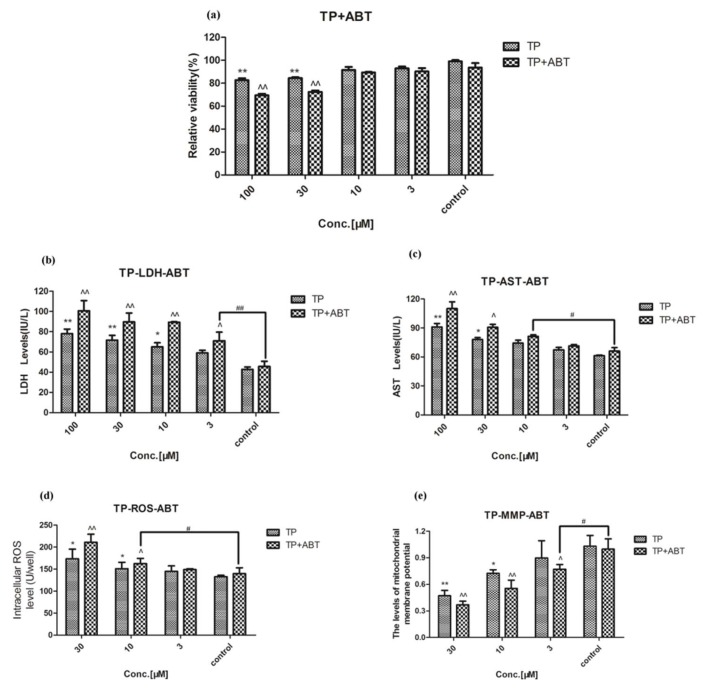
The effect of triptolide on cell after preincubation with ABT. (**A**) Relative viability after treatment with a variety of concentrations of triptolide. (**B**–**D**) Productions of LDH, AST, and ROS in primary rat hepatocytes after exposure to triptolide, respectively. (**E**) MMP levels of primary rat hepatocytes after treatment with different concentrations of triptolide. Compared with the control group, * *p* < 0.05, and ** *p* < 0.01; compared with triptolide group, ^ *p* < 0.05, and ^^ *p* < 0.01; compared with ABT treated group, ^#^ *p* < 0.05, and ^##^ *p* < 0.01.

The activity of CYP450s in the liver cells was inhibited, and the xenobiotic metabolizing capacity was weakened after adding the broad-spectrum inhibitor of CYP450s, ABT, into primary rat liver cells. After preincubating with ABT and treatment with various concentrations of celastrol, relative cell viability had a 10% decrease when compared with the group which was treated with celastrol only (Figure 4A). LDH, AST, and ROS productions were increased significantly (*p* < 0.05) compared with the celastrol group (Figure 4B–D). They were shown in a dose-dependent manner. Δ*Ψm* was decreased after exposure to celastrol (Figure 4E). The results of treatment with triptolide and ABT on XTT, LDH, AST, ROS, and MMP were similar to that of celastrol (Figure 5). Damage to primary rat hepatic cells caused by these two compounds was increased by showing stronger cell viability inhibition, elevated liver function indicators, and more damage to the cell membrane.

### 2.3. Effect of Celastrol and Triptolide on Primary Rat Hepatocytes Model with PB

**Figure 6:**
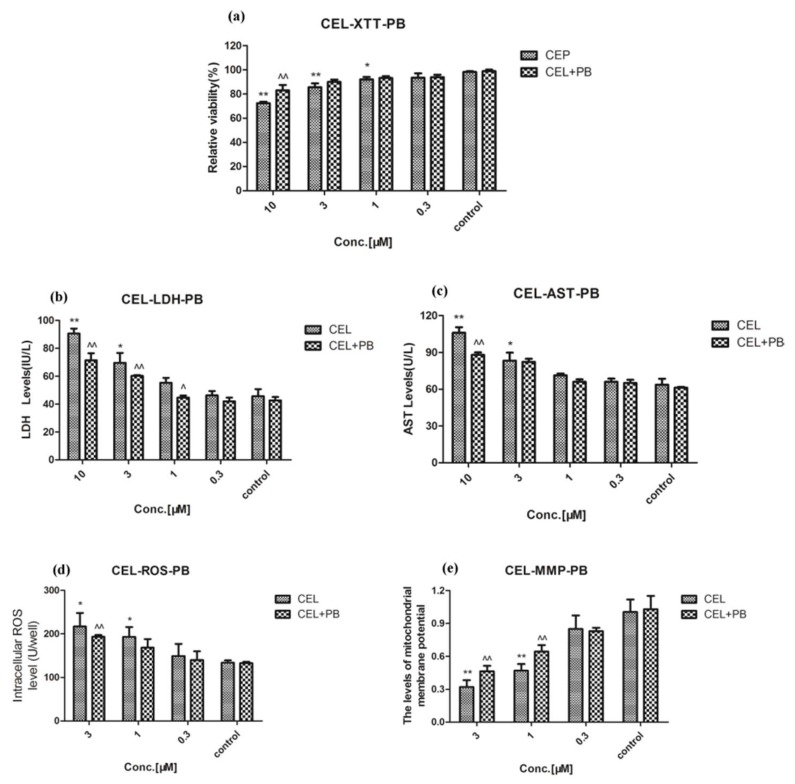
The influence of celastrol on cells after PB was treated at the concentration of 40 mg/kg for five days. (**A**) The relative viability after treatment with a variety of concentrations of celastrol. (**B**–**D**) LDH, AST, and ROS in cellular supernate accumulation in treatment with primary rat hepatocytes, respectively. (**E**) The MMP after exposure to different concentrations of celastrol. Compared with the control group, * *p* < 0.05, and ** *p* < 0.01; compared with celastrol group, ^ *p* < 0.05, and ^^ *p* < 0.01.

**Figure 7:**
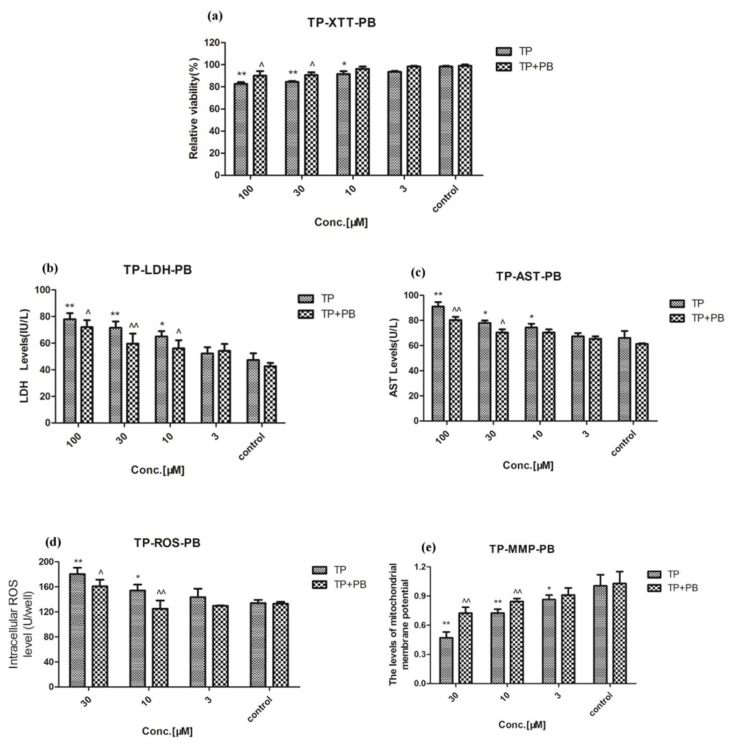
The influence of triptolide on cells after PB was treated at 40 mg/kg for five days. (**A**) Relative viability after treatment with a variety of concentrations of triptolide. (**B**–**D**) Cellular LDH, AST, and ROS accumulations in treatment with primary rat hepatocytes, respectively. (**E**) The MMP after treatment with different concentrations of triptolide. Compared with the control group, * *p* < 0.05, ** *p* < 0.01; compared with triptolide group, ^ *p* < 0.05, ^^ *p* < 0.01.

The activity of CYP450s in the primary rat liver cell model was enhanced through inducing high expression of CYP450s with phenobarbital, especially CYP3A4 (Figure S1). Phenobarbital could stimulate the activity of CYP450s in hepatocytes and intensify its substrate metabolism. Compared with control group, after rats were treated with phenobarbital for five days and administration of various concentrations of celastrol, cell viability and Δ*Ψm* were still decreased significantly (*p* < 0.01) (Figure 6A,E) and levels of LDH, AST, and ROS were increased significantly (*p* < 0.01) (Figure 6B–D). However, compared with celastrol group, the results exhibited a significant reversing effect on the decrease in cell viability and Δ*Ψm* (Figure 6A,E). Levels of LDH, AST, and ROS were decreased significantly through the reversing effect (*p* < 0.01) (Figure 6B–D). For triptolide, similar results also appeared after exposure to triptolide and PB on XTT, LDH, AST, ROS, and MMP (Figure 7). Hepatotoxicity induced by celastrol and triptolide was improved when cells were treated with PB.

### 2.4. Drug-Drug Interaction between Celastrol and Triptolide

**Figure 8:**
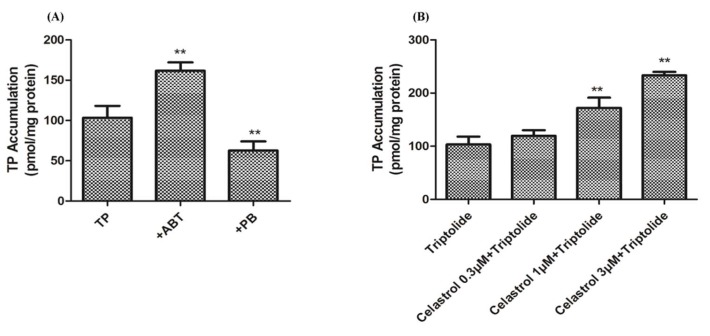
The amount of triptolid in rat primary hepatocyte. (**A**) Accumulation of triptolid in rat primary hepatocyte after treatment with ABT/PB. (**B**) Amount of triptolid in rat primary hepatocyte after exposure to a series of concentrations of celasreol. Compared with triptolid group, ** *p* < 0.01.

**Figure 9:**
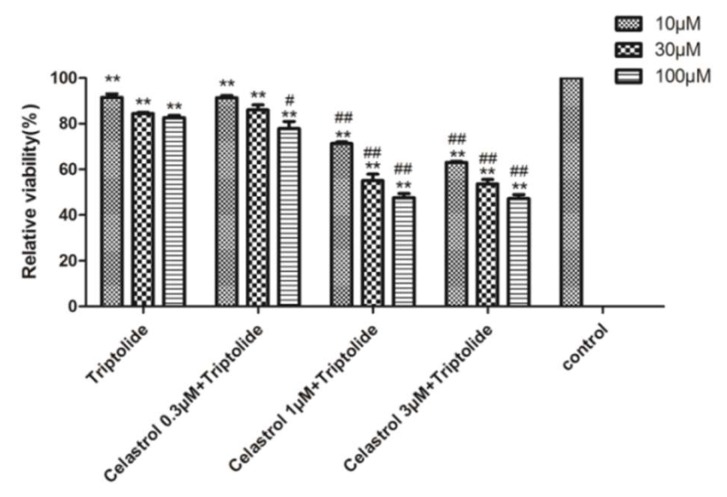
The relative viability after cells were treated with celastrol and triptolid. Compared with the control group, a combination of the two compounds caused cell viability to significantly decrease in a dose-dependent manner; ** *p* < 0.01. Compared with triptolid group, ^#^ *p* < 0.05, and ^##^ *p* < 0.01.

The amount of triptolide in the supernatant was analyzed by LC-MS/MS (Figure S2). Compared with the triptolide group, the amount of triptolid increased significantly (*p* < 0.01) after treatment with ABT. The group treated with PB exhibited a significant (*p* < 0.01) reversing effect on the increase in the amount of triptolid (Figure 8A). The group of triptolid treated with different concentrations of celastrol (0.3, 1, 3 μM) significantly (*p* < 0.01) increased accumulation of triptolid in a dose-dependent manner, as shown in Figure 8B. Especially, when combined with celastrol (0.3, 1, 3 μM) and triptolide (10, 30, 100 μM), compared with the control group and the triptolide group respectively. Cell viability was decreased significantly (*p* < 0.01), both of the two results were in a dose-dependent manner (Figure 9).

## 3. Discussion

The liver plays an important role in biotransformation which is often used to research the metabolic mechanism and metabolic status of a variety of drugs in vitro. In the process of metabolic transformation of drugs in vitro, primary hepatocyte mostly maintains the metabolic function of the liver, keeps a CYP450s level that is consistent with in vivo level, and particularly has great reproducibility. Therefore, the in vitro study with primary hepatocyte provides a criterion for safety evaluation, identifying specific metabolic pathways and a toxicological study of drugs, especially those drugs with low metabolic transformation in vivo, high toxicity, and limited detection methods. In our study, both celastrol and triptolide proved toxic potencies on rat primary hepatocyte. With regard to relative viability, celastrol is much more potent than triptolide, with only ~60% of the cells being viable after treatment with a concentration of 30 μM, whereas ~90% survive after treatment of 30 μM triptolide (Figure 2 and Figure 3).

In the study, we selected five important CYP450 hypotype enzymes in the liver as the objects. However, it is undeniable that we have neglected other P450 hypotype enzymes with pivotal roles, for instance, CYP1A1, CYP2C8, and CYP2B6, etc. Reports have shown that the level of CYP1A1 is an effective indicator to measure the chemical carcinogenicity and characterized by obvious gene polymorphism. Although accounting for less than 1% of CYPs, it is involved in 2.5% drug metabolism [31]. Despite a small content in human liver, CYP2B6 features huge potential for variation. The individual content of CYP2B6 fluctuates between 2 and 82 pmol/mg [32]. We have discovered that celastrol is similar to the structure of acyl-glucuronide, which is an inhibitor of CYP2C8 [33]. As currently there haven’t been definite reports on the metabolism of celastrol via the liver and the metabolites, this will be topped on the list of our next experimental agenda. To enrich and perfect the experimental results, we will further study the characteristics of those hypotype enzymes, which have been left neglected in this experiment.

In some in vitro experiments, the selected experimental concentrations for celastrol range from 0.1 μM–10 μM. In this paper, concentrations of celastrol are identical to those reported in previous studies. Under normal circumstances, celastrol is used as a main component of the Chinese patent medicine *Tripterygium* tablet, with the average content of 236 μg in each tablet [7]. PB could stimulate the activity of CYP450s in hepatocytes and improve its function through inducing high expression of CYP450s [34]. When the same dose was administrated (10 μM celastrol), those cells treated with the inducer of CYP450s showed significantly higher cell viability and different biochemical indicators compared with those cells that were not treated. It suggested that liver toxicity caused by celastrol was reduced in this case.

The metabolic capacity of CYP450s to celastrol is one of the important factors that affect the hepatotoxicity. That is to say, the hepatotoxicity caused by celastrol is directly related to its accumulation in the liver cells. To be an inhibitor of CYP450s [35], celastrol could worsen hepatotoxicity caused by triptolide. As mentioned before, triptolide (not more than 10 μg) is used as the quality control and limited indicator for *Tripterygium polycoride* tablets, due to the presence of plentiful celastrol, it could lower the minimum concentration of triptolide that leads to decreased cell viability. This might be the reason for more severe liver damage caused by the combination of celastrol and triptolide.

## 4. Materials and Methods

### 4.1. Materials

Celastrol, triptolide, and phenobarbital (PB) with a purity of >98% were purchased from the Tianjin YiFang Technologies Co Ltd. (Tianjin, China). 1-aminobenzotriazole (ABT) was purchased from Tokyo Chemical Industry Co Ltd. (Tokyo, Japan), XTT was bought from Sangon Biotech Company (Shanghai, China). Recombinant human cytochrome P450 enzymes (CYP1A2, CYP2C11, CYP2D1, CYP2E1 and CYP3A4) were bought from Reid Ltd., Research Institute for Liver Diseases (Shanghai, China, Batch, No: SUBK). Buspirone was obtained from the National Institutes for Food and Drug Control (Tianjin, China). Collagen, type I solution from rat tail was purchased from Sigma (St. Louis, MO, USA), fetal bovine serum (FBS), and streptomycin-penicillin (S-P), 0.25% trypsin-ethylenediaminetetraacetic acid (EDTA) were obtained from Gibico (Carlsbad, CA, USA). Dulbecco’s modified Eagle’s medium (DMEM) was bought from Hyclone Co. (America). Dimethyl sulfoxide (DMSO) was purchased from Sigma Chemical Co. (St. Louis, MO, USA). The reactive oxygen species (ROS) assay kit was obtained from Nanjing Jiancheng (Nanjing Jiancheng Bioengineering Institute, China). Mitochondrial membrane potential (MMP) assay kit with JC-1 was purchased from Beyotime (Beyotime Institute of Biotechnology, Shanghai, China). Methanol and acetonitrile were high-performance liquid chromatography-grade from Concord Corporation (Tianjin, China). All other regents were of analytical grade.

### 4.2. Animals

All procedures involving animals were conducted in conformity with the Animal Research: Reporting In Vivo Experiments (ARRIVE) guidelines, and were approved by the Academy of Military Medical Science Institutional Animal Care and Use Committee (Certificate No. SCXK620076004). Male Wistar adult healthy rats (weighing 200–220 g), purchased from Huafukang Laboratory Animal Technology Co. Ltd. (Beijing, China) were kept in a controlled environment (temperature 22 °C ± 3 °C, relative humidity 65% ± 10%, and 12 h light/dark cycle) and fed with a standard laboratory diet.

### 4.3. Primary Rat Hepatocytes Model for Evaluating the Toxicity of Celastrol and Triptolide

#### 4.3.1. Isolation and Culture of Hepatocyte

After fasting for 12 h with free access to water prior to the experiment, the rat was anesthetized with chloral hydrate before surgical procedures. Isolated hepatocytes were prepared by collagenase perfusion as previously published [36]. Cells were suspended in high-glucose Dulbecco’s Modified Eagle’s Medium (DMEM) supplemented with 10% fetal bovine serum, 100 U/mL penicillin, and 100 μg/mL streptomycin. After been determined by the 0.4% trypan blue dye exclusion test, hepatocytes with viability of greater than 95% were used. Cells were routinely plated at a density of 1.5 × 10^4^ cells/well in 96 well plate precoated with rat tail collage. All plates were incubated at 37 °C and 5% CO_2_ in a humidified atmosphere for 4 h.

#### 4.3.2. XTT Assay

XTT is a widely used test method to measure cell viability and proliferation. In the study, after washing twice with warm phosphate buffer saline (PBS), cells were incubated with celastrol (0.3, 1, 3, 10 and 30 μM) and triptolide (1, 3, 10, 30, 100 and 300 μM) for 24 h at 37 °C, respectively. Then XTT-PMS (50:1) solution was added in each well. Absorbance value at 450 nm was measured in a molecular device (Flex Station 3, Molecular Devices, Silicon Valley, CA, USA) after incubation for 4 h. The relative cell viability was determined by the ratio (%) of the absorbance of treated cells to that of untreated cells. Each test condition was analyzed in triplicate.

#### 4.3.3. The Determination of LDH and AST Level

As important indexes to evaluate hepatocyte injury, lactic dehydrogenase (LDH) and glutamic-oxalacetic transaminease (AST) release in culture medium (96-well plate) were measured after cells were treated with celastrol (1, 3, 10, and 30 μM) and triptolide (3, 10, 30, and 100 μM) for 24 h, respectively. Results are expressed as international unit per liter (IU/L).

#### 4.3.4. Detection of ROS Generation

The production of ROS was determined by detecting the fluorescent probe 2′, 7′-dichlorofluorescin diacetate (DCFH-DA). Hepatocytes in the plates were incubated for 24 h after administration of celastrol (1, 3 and 10 μM) and triptolide (3, 10 and 30 μM) respectively. Cells were treated continually with 100 μL of 20 μM DCFH-DA for 40 min at 37 °C. They were washed twice with PBS and were added 100 μL medium. The fluorescence was measured by a molecular device (Flex Station 3, Molecular Devices, Silicon Valley, CA, USA) at an excitation wavelength of 485 nm and an emission wavelength of 528 nm.

#### 4.3.5. The Determination of MMP

Mitochondrial membrane potential (*ΔΨm*) was monitored with JC-1, which preferentially localizes to active mitochondria based on highly negative Δ*Ψm*. After the treatment with celastrol (1, 3 and 10 μM) and triptolide (3, 10 and 30 μM) for 24 h respectively, JC-1 was added to each well and incubated for 20 min at 37 °C. Then cells were washed twice by JC-1 staining buffer and analyzed by fluorospectrophotometry at monomers (λ_ex_ 514 nm and λ_em_ 529 nm) and aggregates (λ_ex_ 585 nm and λ_em_ 590 nm). The *ΔΨm* of cells in each treatment group was calculated as the fluorescence ratio of monomers to aggregates. Mitochondrial depolarization will be shown by a decrease in the monomers/aggregates fluorescence intensity ratio.

### 4.4. Evaluate Toxicity of Celastrol and Triptolide on Primary Rat Hepatocytes with Subdued Enzyme Activity

1-aminobenzotriazole (ABT), as an extensively nonspecific and effective inhibitor of CYP450 enzymes [37,38], is thought to inactivate CYP450s following complex formation with metabolic intermediates (MI) [39]. Cells in the plate were preincubated with ABT (0.5 mM) for 30 min, after washing twice by PBS, they were incubated with celastrol (0.3, 1, 3, and 10 μM) or triptolide (3, 10, 30, and 100 μM) with/without ABT for 24 h, respectively. The methods of determining XTT, ROS, AST, LDH, and MMP level are mentioned as above.

### 4.5. Evaluating the Toxicity of Celastrol and Triptolide on Primary Rat Hepatocytes with Enhanced Enzyme Activity

Phenobarbital (PB), as the most widely used antiepileptic drug in the clinical, is known as an inducer of CYP450 enzymes which has the responsibility of inducing the gene expression of CYP450s [40]. Primary rat hepatocytes model with enhanced activity CYPs was used by previous studies as described [41], then cells were incubated with celastrol (0.3, 1, 3 and 10 μM) or triptolide (3, 10, 30 and 100 μM) at 37 °C for 24 h and level of XTT, ROS, AST, LDH and MMP were measured as described above, respectively.

### 4.6. Cell Amount of Triptolide Assay

The procedure for treatment of primary rat hepatocytes with ABT/PB was as described above after treatment with 10 μM triptolide for 24 h and washed three times by ice-HBSS, cells in the plates were disrupted in HBSS, 150 ng/mL buspirone as the standard solution and 0.1 M formic acid were added in each well. Samples were abstracted by ethyl acetate for 10 min. After centrifugation at 17,000 rpm for 10 min, the supernatant was collected and exsiccated by termovap sample concentrator. After that, samples were dissolved in 100 μL methyl alcohol-water (1:1) and centrifuged at the same condition, the amount of triptolide in supernatant was analyzed by API 4000 Qtrap UPLC-MS/MS (Applied Biosystems, America). The same assay was used to study amount of triptolide in hepatocytes by replacing ABT/PB with celastrol (0.3, 1, 3 μM). Cell relative viability (%) was also measured after the combination of triptolide (10, 30 and 100 μM) and celastrol (0.3, 1 and 3 μM).

### 4.7. LC-MS/MS Analysis

The LC/MS/MS method was conducted as described in Reference [12]. The method was carried out using a Waters Acquity UPLC Sample Manager and a Waters Acquity UPLC Binary Solvent Manager connected to a Waters Quattro Premier XE triple-quadruple mass spectrometer and Mass Lynx 1.5.2 software (Waters, Milford, MA, USA). An Agilent Zorbax XDB-C_18_ (3.5 mm, 2.1 mm × 50 mm) was also used. The gradient mode was used to achieve triptolide and internal standard using mixtures of 40 mM ammonium formate (mobile phase A) and methanol with 0.1% (*v*/*v*) formic acid (mobile phase B) at a flow rate of 0.5 mL/min. Mobile phase A was 98% at 0.1–0.6 min, then a linearly programmed gradient from 98% to 5% at 3–4.5 min, at last, mobile phase A ramp to 98% at 4.5–6 min toward the end of the analysis. The system was auto-injected with 10 μL of each analyte. Multiple reaction monitoring (MRM) mode was applied to achieve quantification with monitoring precursor-product ion transitions of *m*/*z* 361.0→105.0 for triptolide using electrospray ionization mass spectrometry (ESI-MS) on an API-4000, with Turbo Ionspray. The system was in positive ESI-mode during the run. The desolvation temperature was set at 550 °C and the nebulizer gas pressure was 60 psi.

### 4.8. Statistical Analysis

Each sample was run in triplicate. The results of the XTT, LDH, AST, ROS, and MMP assays were shown as a mean ± standard deviation (SD) and analyzed by GraphPad Prism (version 5.0) software (Graphpad Software, San Diego, CA, USA). For data of biomarkers, comparisons between two groups were made by independent sample t-tests. SPSS 11.5 was used for statistical analyses. Differences were considered significant when the calculated *p* < 0.05 or *p* < 0.01.

## 5. Conclusions

In summary, liver injury caused by celastrol and triptolide in primary rat liver cells was reflected by reducing cell viability, increasing LDH and ROS levels, decreasing MMP, enhancing cellular stress, and a certain degree of damage on the cell membrane. The ingredient with hepatotoxicity in *Tripterygium wilfordii* might be celastrol, which is closely associated with the activity of CYP450 metabolic enzymes in the liver. Nevertheless, hepatotoxicity of triptolide is caused by the amount of itself, which means liver damage becomes worse and more easily affected by other drugs (inhibitors of CYP450s) along with its accumulation. Celastrol could inhibit the activity of CYP450s in the liver and lead to potential drug–drug interactions in pharmacodynamics and pharmacokinetics. Due to its immunosuppressive activity, anti-tumor activity, and potential hepatotoxicity, much attention must therefore be observed to avoid this.

## Supplementary Materials

The Supplementary Materials are available online.

## Abbreviations

CYP450	Cytochrome P450
XTT	2,3-Bis-(2-methoxy-4-nitro-5-sulfophenyl)-2H- tetrazolium-5-carboxanilide
LDH	lactic dehydrogenase
AST	aspartate aminotransferase
ROS	reactive oxygen species
MMP	mitochondrial membrane potential
ABT	1-aminobenzotriazole
PB	phenobarbital
FBS	fetal bovine serum
S-P	streptomycin-penicillin
EDTA	trypsin-ethylenediaminetetraacetic acid
DMSO	dimethyl sulfoxide
DMEM	Dulbecco’s modified Eagle’s medium
PBS	phosphate buffer saline
DCFH-DA	2′, 7′-dichlorofluorescin diacetate
FDA	food and drug administration
MI	metabolic intermediate
MRM	multiple reaction monitoring
ESI-MS	electrospray ionization mass spectrometry
SD	standard deviation
