# Supplementary material for: CYP450s-Activity Relations of Celastrol to Interact with Triptolide Reveal the Reasons of Hepatotoxicity of Tripterygium wilfordii
Source: Molecules. 2019 Jun 8;24(11):2162. doi: 10.3390/molecules24112162 (PMC6600472; doi:10.3390/molecules24112162)
Supplement: Supplementary file 1 [file molecules-24-02162-s001.pdf]

Supplementary Materials

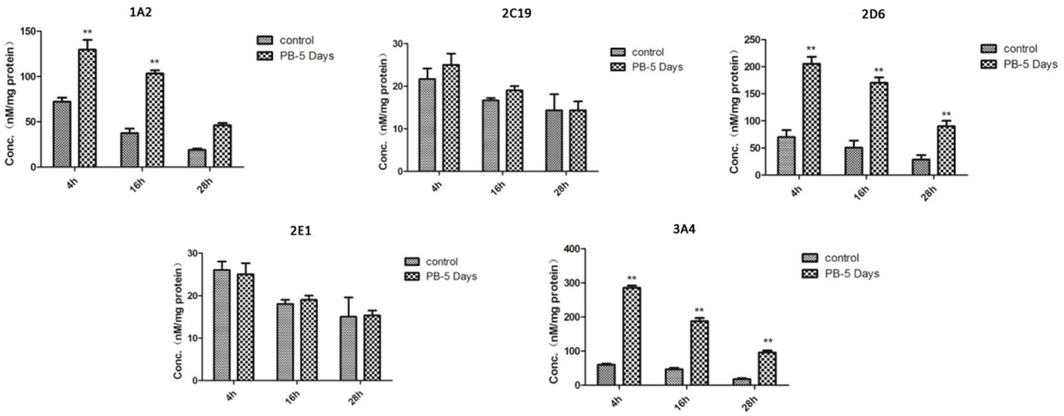

Figure S1. The amount of metabolites of five specific substances (phenacetin, mephenytoin, dextromethorphan, chlorzoxazone, midazolam) of CYPs (1A2, 2C19, 2D6, 2E1, 3A4) in primary rat hepatocytes. Compared with control group, the amount of metabolites of five specific substances in CYP1A2, CYP2D6, CYP3A4 is increased significantly, **p<0.01.

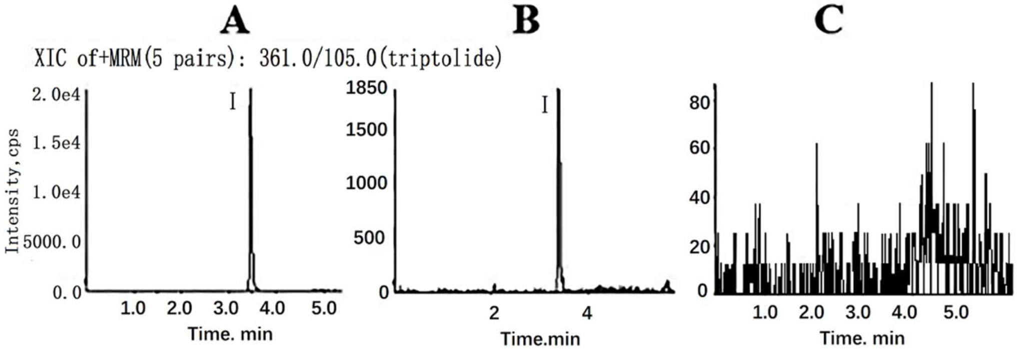

Figure S2. UPLC-MS/MS chromatograms of triptolide derived from methyl alcohol (**A**), DMEM (**B**) and hepatocyte samples (**C**).
